# Five-Year-Olds’ and Adults’ Use of Paralinguistic Cues to Overcome Referential Uncertainty

**DOI:** 10.3389/fpsyg.2018.00143

**Published:** 2018-02-13

**Authors:** Justine M. Thacker, Craig G. Chambers, Susan A. Graham

**Affiliations:** ^1^Owerko Centre and Department of Psychology, University of Calgary, Calgary, AB, Canada; ^2^Department of Psychology, University of Toronto Mississauga, Mississauga, ON, Canada

**Keywords:** talker preference, speech disfluencies, eye tracking, spoken language comprehension, referential intent, pragmatic inference

## Abstract

An eye-tracking methodology was used to explore adults’ and children’s use of two utterance-based cues to overcome referential uncertainty in real time. Participants were first introduced to two characters with distinct color preferences. These characters then produced fluent (“*Look! Look at the blicket.*”) or disfluent (“*Look! Look at thee, uh, blicket.*”) instructions referring to novel objects in a display containing both talker-preferred and talker-dispreferred colored items. Adults (Expt 1, *n* = 24) directed a greater proportion of looks to talker-preferred objects during the initial portion of the utterance (“*Look! Look at*…”), reflecting the use of indexical cues for talker identity. However, they immediately reduced consideration of an object bearing the talker’s preferred color when the talker was disfluent, suggesting they infer disfluency would be more likely as a talker describes dispreferred objects. Like adults, 5-year-olds (Expt 2, *n* = 27) directed more attention to talker-preferred objects during the initial portion of the utterance. Children’s initial predictions, however, were not modulated when disfluency was encountered. Together, these results demonstrate that adults, but not 5-year-olds, can act on information from two talker-produced cues within an utterance, talker preference, and speech disfluencies, to establish reference.

## Introduction

Imagine that a mother and her preschooler are baking a cake, and the mother instructs her child to “*Pass the spatula!*” How might the child, who does not know what a spatula is, identify the intended referent from among the many possible kitchen objects that are unfamiliar? Fortunately, various speaker-produced behaviors provide cues that help young word learners identify the intended referent of a novel word, including eye gaze direction (e.g., Baldwin, 1991, 1993; Graham et al., 2010), gestures (e.g., O’Neill et al., 2002), facial expressions (e.g., Akhtar and Tomasello, 2000; Henderson and Graham, 2005; Graham et al., 2006), and emotional prosody (Berman et al., 2013a). Spoken language, however, also contains signals that are not a direct expression of communicative intent yet nonetheless can serve as cues for establishing referential mappings. Here, we examine the potential contribution of two such cues to referential intent, namely speech disfluencies and cues to talker identity that evoke knowledge of talkers’ preferences. Specifically, we explore adults and 5-year-olds’ ability to coordinate their knowledge of talkers’ preferences with the fluency of their speech to determine reference.

As noted above, one candidate cue for word learning is talker preference. Previous studies have demonstrated that children understand the link between a speaker’s explicitly stated desire for an object and an object that is then chosen (Wellman and Bartsch, 1988; Wellman and Woolley, 1990; Repacholi and Gopnik, 1997; Saylor and Troseth, 2006; Fawcett and Markson, 2010), and can use this link to facilitate word learning. For example, Saylor and Troseth (2006) introduced 3-year-olds to pairs of novel toys with an experimenter indicating which toy she preferred (e.g., “*I like this one!*”). Next, when the researcher used a novel noun to express her desire to play with one of the toys (e.g., “*I really want to play with the riff.*”). Children accurately selected the experimenter-preferred toy from the array, even when it conflicted with the child’s own preference. In a related line of research, studies have demonstrated that preschoolers can use their knowledge of talker preferences to guide real-time referential processing (e.g., Creel, 2012, 2014; Borovsky and Creel, 2014). In one such study, Creel (2012) introduced children to two characters, each with a distinctly gendered voice (i.e., male and female) and each having a preferred color (i.e., blue and pink). Following the introductions, when children heard the onset of either talker’s voice, they demonstrated anticipatory looking to visually displayed shapes bearing that talker’s preferred color. (Note that the characters themselves were no longer depicted at this point.) Thus, children drew upon a set of associations (acoustic voice characteristics → talker → preferences) to help identify relevant referents in real time. Children’s use of talker preferences is, however, flexibile. In a second experiment, children not only used talker identity cues to anticipate reference to talker-preferred objects when characters were speaking on their own behalf, but also accurately identified relevant referents when one character made a request on behalf of the other character. Relatedly, Borovsky and Creel (2014) demonstrated that 3- to 10-year-old children, like adults, generate similar expectations when the associations involve generic knowledge instead of explicit preference information. For example, if a talker is introduced as a “pirate,” children will predict that this talker is more likely to request a sword than a magic wand, even though the talker hadn’t mentioned these objects or a preference for objects with certain kinds of properties. As before, referential predictions were generated quickly, simply upon hearing the talker’s voice.

Here, we ask whether talker preferences, and their association with a talker’s voice, can function as an effective cue to overcome referential uncertainty. Specifically, it remains unclear whether preschoolers can accurately identify speaker-preferred referents on the basis of a property (such as color) when the object itself is unfamiliar. Given preschoolers’ skilled use of talker identity to predict which familiar object a speaker will refer to, it is conceivable that preschoolers can link a speaker’s referential intent to novel objects on the basis of a preferred property. However, it is also possible that the unfamiliarity of the objects could overshadow attention to nonfunctional properties such as color, making it less likely that children will make referential links to novel objects simply on the basis of preference information. It is also unclear whether a desire for preferred objects needs to be linguistically communicated for talker preference information to be activated. Most research to date, for example, has included desire statements (e.g., “*I want*…”) that provide an explicit signal that a talker’s preferences are relevant to the communicative context. Knowledge about preferences, however, could be relevant to referential intentions even when desire is not explicitly voiced. Thus, one goal of the present study was to determine whether listeners can use knowledge about talker preferences, in the absence of explicit desire statements, to facilitate their interpretation of novel words.

A second goal was to examine listeners’ sensitivity to and use of two utterance-based cues occurring in the same utterance. In order to address this question, we use a second incidentally produced and talker-specific cue, namely momentary speech disfluencies. Research to date has shown that even young children can use common minor speech disfluencies such as filled pauses (e.g., “*um*”/“*uh*”) to anticipate reference to certain types of entities. In the first such study, Kidd et al. (2011) presented toddlers with familiar-novel object pairs (e.g., a ball and a gorp). On each trial, the familiar object was labeled in order to establish that object as discourse-given. On the third trial, children heard an instruction containing a fluent or disfluent description of one of the objects. Disfluent instructions led 30-month-olds to anticipate reference to the novel discourse-new object. As the discourse-new object was also always the novel object in the Kidd et al. (2011) paradigm, a recent series of studies sought to disentangle these factors. These findings demonstrated that 2- and 3-year-old children readily associate filled pauses with upcoming reference to discourse-new (Owens and Graham, 2016) objects but not unfamiliar objects (Owens et al., 2017). This contrasts with studies showing that adults show referential anticipation of novel objects upon hearing a filled pause (Arnold et al., 2007; Owens et al., 2017). Recently, research has highlighted preschoolers’ ability to amend an initial prediction (e.g., that talkers are more likely to refer to a preferred familiar object) when encountering a disfluency (Thacker et al., 2018). For the present study, we sought to explore whether this ability can also support preschoolers’ formation of referential predictions for novel objects. To this end, we asked whether disfluencies would similarly lead children to amend an initial prediction that talkers are more likely to refer to a preferred object over a dispreferred object.

In summary, previous research has established that children can use cues based on talker identity and talker preferences as well as disfluency cues to guide initial referential mappings. Here, we explore whether and how children are sensitive to and draw on information from distinct paralinguistic cues within an utterance during reference resolution, as real-world situations are likely to contain multiple cues that may or may not necessarily point to the same referential candidate. Thus, the goal of the present experiments is to examine adults’ and 5-year-olds’ use of recently presented preference information together with speech fluency to guide referential predictions for novel objects.

Following work by Creel (2012), we used a paradigm where two characters, a male and a female, were first introduced to participants and described as having opposite color preferences (i.e., blue and pink). In addition, the characters established a tendency to refer to novel objects of their preferred color, thus providing evidence for the described preferences. In the second phase of the experiment, listeners were presented with displays consisting of two novel objects, one pink and one blue, accompanied by an utterance directing listeners to look and point at one of the objects using a novel description that was in a fluent (e.g., “*Look at the blicket. Point to the blicket.*”) or disfluent (“*Look at thee, uh, blicket. Point to the blicket.*”) utterance. During the initial portion of the utterance, we focused particularly on whether children would draw on the established associations between talkers and preferred objects. If so, this would likely lead them to expect a talker-preferred object as the intended referent. We also examined whether children understand the filled pause occurring in the same instruction as a source of evidence that *attenuates* the link between speakers and novel objects bearing preferred properties. More specifically, will disfluency shift consideration toward novel entities that are less contextually probable given the talker’s identity (i.e., objects *not* bearing the talker’s preferred color)?

## Experiment 1

We tested adults in Experiment 1 to evaluate how mature language users would integrate knowledge of talkers’ preferences with the fluency of talkers’ speech to overcome referential uncertainty in the context of novel words. This data provided an important benchmark for children’s results.

### Materials and Methods

#### Participants

Data from 23 university students (seven males; *M*_age_ = 21.40 years, *SD* = 4.34 years) were included in the final sample. Five additional participants were tested but excluded from the analyses as no target fixations were registered for any trial in at least one of the experimental conditions. Participants were primarily of European-Canadian heritage and reported English as their primary spoken language. Informed written consent was obtained for each participant.

#### Materials and Apparatus

**FIGURE 1:**
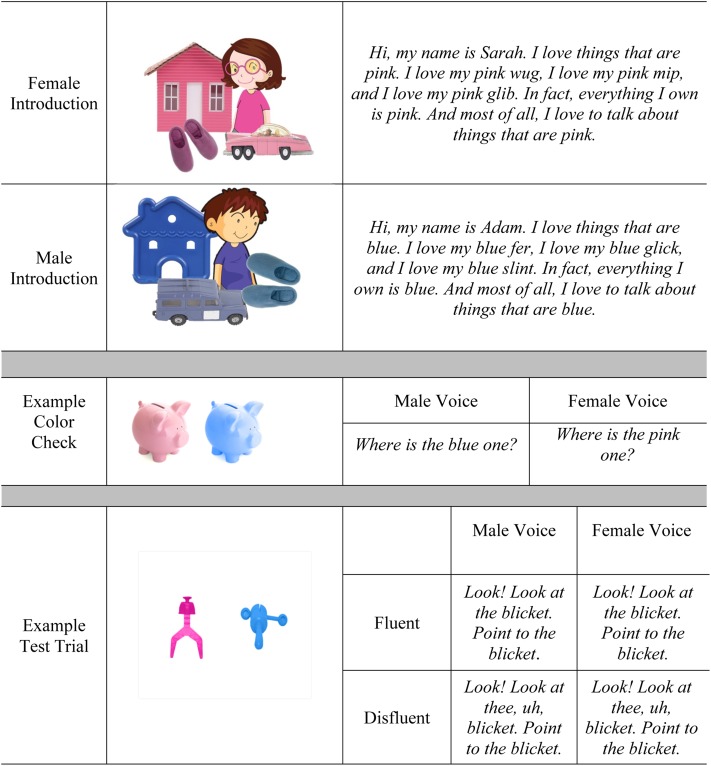
Testing sequence with examples of visual and auditory materials. Images are adapted from “Clipart.com” (http://www.clipart.com). Copyright 2018 by Vital Imagery Ltd. Reprinted with permission.

The visual stimuli on critical trials consisted of 16 photographs of pink novel objects and 16 photographs of blue novel objects, combined to make 16 pink/blue-novel object pairs. Two native speakers of English, one female and one male, recorded the auditory stimuli. We chose to use a male and a female voice as listeners may have difficulty distinguishing between same-gender talkers due to their acoustic similarity (Creel and Jimenez, 2012). Talkers first recorded an introduction for their character (see **Figure 1** for the script used in the voice introduction phase). The introductions depicted each character on the screen and established that each character had a preferred color (i.e., pink and blue), and that they labeled unfamiliar objects in their preferred color. After the introductions, the depictions of the two characters were no longer displayed. Eight alternating color-check trials followed the introductions. The male talker gave instructions on four of the color-check trials (“*Where is the blue one?*”) and the female talker gave instructions on the other four trials (“*Where is the pink one?*”). These trials allowed us to verify that each participant could distinguish the colors, and reinforced participants’ knowledge of the characters’ color preferences. Then, for each of the 16 object pairs, each talker recorded a fluent and disfluent version of the critical instruction to look at an object labeled by a novel noun (“*Look! Look at the X.*”and “*Look! Look at thee, uh, X.*”), as well as an instruction to point at the object (“*Point to the X.*”). In all cases, a novel noun phrase was used (e.g., “…*the wug.*”). To provide more detailed insight into how listeners rely on cues to form referential predictions, we used two different measures. First, we recorded listeners’ points toward selected objects as a marker of their final referential decision. Second, we recorded participants’ eye gaze throughout the unfolding utterance to provide a marker of their real-time consideration of referential alternatives. Although these two measures can reflect distinct processes, we predicted that the implicit and more fine-grained measure of processing provided by participants’ eye gaze would be related to listener’s explicit selection of a referent while pointing.

To ensure consistency across test trials, particular segments of the recorded utterances were standardized. Specifically, the initial “*Look! Look at*” was excised from the same recording and then spliced into all fluent trials. In addition, the same disfluent portion of the utterance, “*Look at thee, uh…*”, was excised and spliced into all disfluent trials. Utterances were recorded in their entirety and were edited using Audacity, a multi-track audio editor. There were four trials in each of the four patterns that resulted from crossing the female and male talker and fluent versus disfluent utterance manipulations. The pairing of trials to patterns was cycled across participants such that each object pair occurred in all patterns, but each participant saw a given object pair only once.

The visual stimuli were presented to participants on a 46-inch screen. As participants listened to the recordings and viewed the images on the screen, their gaze position was tracked using a TOBII ×50 system with a PC-controlled tracking camera located on a table surface in front of the participant and directly underneath the display screen. The eye tracker sampled participant gaze data every 20 ms. Participants’ eye movements to two areas of interest (AOIs), which were defined prior to testing and corresponded to the location of the two objects on the screen, were tracked and gaze to a particular AOI was registered as a fixation when gaze position was stable for at least 100 ms. For analysis purposes, the gaze data were integrated with the prerecorded utterances using Eye-gaze Language Integration (ELIA) software (Berman et al., 2013b). Calibration of the eye tracker was was conducted using Clearview software and the experiment proper was conducted using E-Prime software.

#### Procedure

To begin, participants were seated in front of the eye tracker and their gaze position was calibrated. Accurate calibration was required on at least three out of the five test fixation points prior to initiating the experiment, with calibration being achieved for all five fixation points for 92% of participants. When the experiment proper began, each participant was presented with the female and the male introductions, followed by eight color check trials, in turn followed by a re-introduction to each character. Next, participants viewed four test trials of each trial type, *Fluent/Female*, *Fluent/Male*, *Disfluent/Female*, and *Disfluent/Male*, presented in a quasi-randomized fashion. Recall that for the test trials, all of the utterances contained referring expressions that used novel nouns and were thus referentially ambiguous.

### Results and Discussion

#### Pointing

Adults’ pointing behavior was used as a measure of their overt ability to detect and combine two paralinguistic cues [i.e., talker preference information and (dis)fluency] with the referring novel noun. Adult pointing data were coded from videotapes by a research assistant who was unaware of the experimental hypotheses and who had no information about the talker or fluency manipulations on a given trial (as all coding was done with no sound playback). A second researcher recoded 20% of the data (five participants) to establish inter-rater reliability. Inter-rater reliability was excellent (Cohen’s Kappa = 1; *p* < 0.001).

We calculated a proportion measure in which adults’ points to the talker-preferred object were divided by the total number of points for fluent trials and disfluent trials. Then, to examine how the choice between the two referents was influenced by the fluency of the utterance, we contrasted scores for fluent trials (*M* = 0.86, *SD* = 0.25) and disfluent trials (*M* = 0.74, *SD* = 0.34). A repeated measures ANOVA did not reveal a significant main effect of fluency (*p* = 0.171). As such, we collapsed the scores across both fluency conditions, and compared the selection of this object to chance (0.50). A one-sample *t*-test indicated that points to the talker-preferred object (*M* = 0.80, *SD* = 0.21) occurred significantly above chance levels (0.50), *t*(22) = 7.001, *p* < 0.001. Thus, across fluency conditions, adults showed a reliable preference to point to the talker-preferred object.

The pointing data suggest that adults’ final selection of a referent for the spoken description was primarily influenced by their knowledge of talkers’ color preferences. Although there was a slight numerical advantage in pointing to the talker-preferred object following fluent utterances, this pattern was not strong enough to entail a significant effect of fluency. Given that eye gaze measures can detect sensitivity to informational cues that may not be fully reflected in listeners’ overt behaviors (Tanenhaus et al., 2000; Berman et al., 2010), we analyzed eye gaze data to gain further insight into the real-time processes that preceded listeners’ explicit responses.

#### Eye Gaze

**FIGURE 2:**
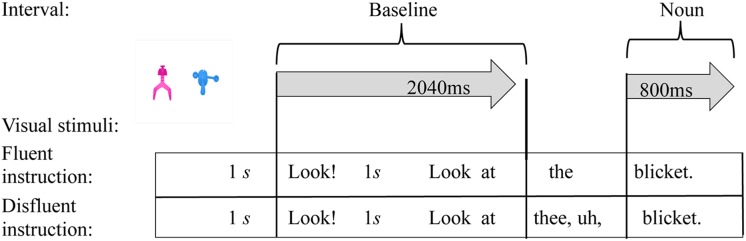
The presentation of the object pairs was followed by an instruction to look at one of the objects with either a fluent or disfluent description. The intervals analyzed were 2040 ms in duration for the baseline region and 800 ms for the noun region.

The measures of interest involve adults’ eye movement behavior at selected time points during the unfolding instructions. The instructions “*Look! Look at the X.*” or “*Look! Look at thee, uh, X*.” were divided into two distinct intervals for analysis (**Figure 2**). To first address whether participants use talker preference information to form predictions about the intended referent as the talker’s voice is initially recognized, we examined fixations during the initial portion of the utterance, which we refer to as the *baseline interval*. We also examined how listeners’ referential expectations were affected by the fluency of the unfolding description. To do so, we calculated fixations in the portion of the utterance that immediately followed the determiner (*noun interval*).

#### Baseline Interval

**FIGURE 3:**
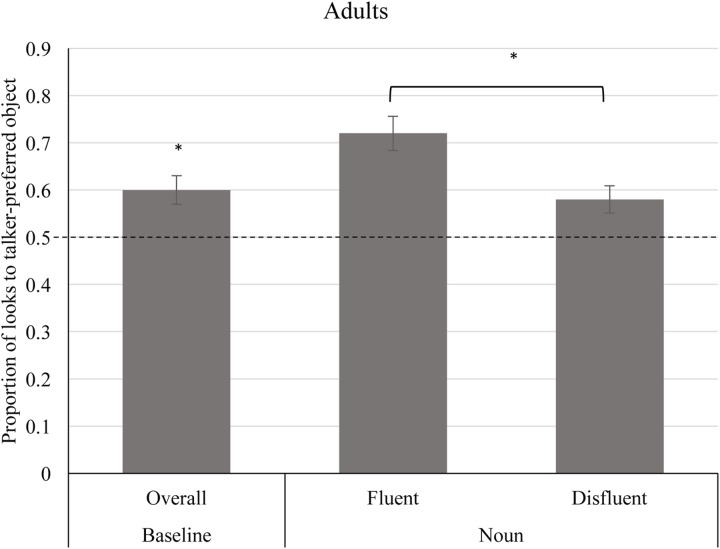
Average proportion of time fixating talker-preferred objects during the baseline interval and noun interval for adult age group. The average proportion of time looking to the talker-preferred object was significantly greater than chance (0.50) during the baseline interval (*p* < 0.001). There was a significant effect of fluency during the noun interval (*p* = 0.041), such that adult participants directed a greater proportion of looking time to the talker-preferred object during the noun interval for fluent trials compared to disfluent trials. Error bars depict standard errors. A single asterisk represents significant differences at the *p* < 0.05 level.

For statistical analysis purposes, we calculated the average proportion of time spent looking to the talker-preferred object (average time spent fixating the talker-preferred object divided by the total time spent fixating both objects). As a result, a value of 1 indicated that the listener only fixated the talker-preferred object during the interval, and a value of 0 means that the listener only fixated the talker-non-preferred object. We then compared the proportion of talker-preferred looking to chance during the baseline interval, which allowed us to evaluate whether adults held an expectation that talkers will refer to objects that are of their previously expressed preferred color. This interval consisted of the shortest duration of the interval corresponding to “*Look! Look at*…” for both the fluent and disfluent utterances (2800 ms), using the endpoint of this window as the boundary point. Results are collapsed across the fluency manipulation within this interval (recall that the information that distinguishes the fluent and disfluent condition is not encountered until after the endpoint of the current analysis interval). **Figure 3** (left column) shows the average proportion of looks to talker-preferred objects during the baseline interval. A one-sample *t*-test indicated that the proportion of time looking to the talker-preferred object (*M* = 0.60, *SD* = 0.15) during the baseline interval was significantly above chance levels (0.50), *t*(23) = 4.05, *p* < 0.001. Thus, adults expected talkers to label objects in their preferred color during the baseline interval.

#### Noun Interval

We next analyzed the effect of the fluency manipulation on the process of mapping the ambiguous noun to a referent. If the presence of a filled pause affects adults’ noun-to-object mappings in the predicted way, we would expect adults to show greater consideration of the talker-preferred object when the utterance was fluent, compared to when the utterance was disfluent, beginning around the time of the determiner. However, the different durations of the determiner across fluent and disfluent trial types ([*the*] = 192 ms; [*thee uh*] = 1862 ms) present a challenge for comparing looking patterns during this region. Thus, to create comparable analysis windows, we analyzed looks immediately following the period of the determiner, beginning with the onset of the noun, for a total duration of 1000 ms. The 1000 ms duration corresponded to the length of the longest noun across trials. For nouns that were shorter in duration, data from the noun interval included some looks during the period of silence that followed noun offset. A 200 ms margin was added to the boundary points of this interval to reflect the time lag in the programming and execution of eye movements (Matin et al., 1993; Allopenna et al., 1998). Again for statistical analysis purposes, we divided proportion of time looking to the talker-preferred object by the sum of looking to both the talker-preferred and talker-non-preferred objects, averaged over the interval. Recall that, a value of 1 indicated that the listener only fixated the talker-preferred object during the interval, and a value of 0 meant that the listener only fixated the talker-non-preferred object.

**Figure 3** (center and right columns) shows the average proportion of time looking to talker-preferred objects in the two fluency conditions. A repeated measures ANOVA revealed a significant effect of fluency, such that adult participants looked less at talker-preferred objects during disfluent utterances (*M* = 0.58, *SD* = 0.23) compared to fluent utterances (*M* = 0.72, *SD* = 0.18), *F*(1,23) = 4.76, *p* = 0.041. Thus, adult participants were more likely to continue fixating the talker-preferred object during fluent trials than during disfluent trials, indicating that disfluency reduced adults’ consideration of the preferred-colored object as the most probable candidate for the novel word. Moreover, whereas looks to the talker-preferred object were significantly greater than chance (0.50) for fluent utterances, *t*(23) = 5.739, *p* < 0.001, this was not the case for disfluent utterances, *p* = 0.065.

The results of Experiment 1 indicated that adults use knowledge of talkers’ color preferences in conjunction with filled pauses to overcome referential uncertainty. Although adults initially relied on preference information when forming referential predictions, they dynamically modified these predictions in the presence of disfluency. The influence of the disfluency cue was, however, only apparent in adults’ implicit referential predictions (as reflected in their eye gaze), not in their explicit referential decisions. This result is in line with previous evidence demonstrating that eye gaze measures can detect sensitivity to informational cues that is not fully reflected in listeners’ overt behavior (Tanenhaus et al., 2000).

## Experiment 2

The aim of Experiment 2 was to determine whether 5-year-olds, like adults, apply talker preference cues and disfluency cues when making predictions about referential intent.

### Materials and Methods

#### Participants

Data from 27 5-year-olds (14 males; *M*_age_ = 5.3 years, *SD* = 0.19 years) were included in the final sample. One additional child was tested but excluded from the analyses as no target fixations were registered for any trial in one of the conditions. Participants were primarily of European-Canadian heritage and English was reported to be the primary spoken language. The majority of parents (96%) reported having at least some post-secondary education. Informed written consent was obtained from the parent of each participant.

#### Materials, Apparatus, and Procedure

The stimuli and procedures were identical to those used in Experment 1. The majority (96%) of child participants achieved calibration for 5/5 calibration points.

### Results and Discussion

#### Pointing

Children’s pointing behavior was coded and analyzed in the same manner described in Experiment 1. Again, inter-rater reliability between the two blind coders was excellent (Cohen’s Kappa = 0.97; *p* < 0.001).

As before, we calculated a proportion measure in which children’s points to the talker-preferred object were divided by the total number of points. We then compared scores on fluent trials (*M* = 0.87, *SD* = 0.22) versus disfluent trials (*M* = 0.88, *SD* = 0.18). A repeated measures ANOVA did not reveal a significant main effect of fluency (*p* = 0.875). As such, we collapsed scores across the fluency conditions, and compared the preference to point to the talker-preferred object relative to chance (0.50). A one-sample *t*-test indicated that points to the talker-preferred object (*M* = 0.88, *SD* = 0.18) were significantly above chance levels (0.50), *t*(26) = 10.937, *p* < 0.001. Thus, like adults, children showed a reliable preference to point to the talker-preferred object.

The pointing data suggest that children’s final selection of a referent for the spoken description was robustly influenced by their knowledge of talkers’ color preferences. However, in order to provide a more fine-grained analysis of children’s processing of these paralinguistic cues, we also analyzed eye gaze data.

#### Eye Gaze

Children’s gaze data were analyzed using the same intervals described in Experiment 1.

#### Baseline Interval

**FIGURE 4:**
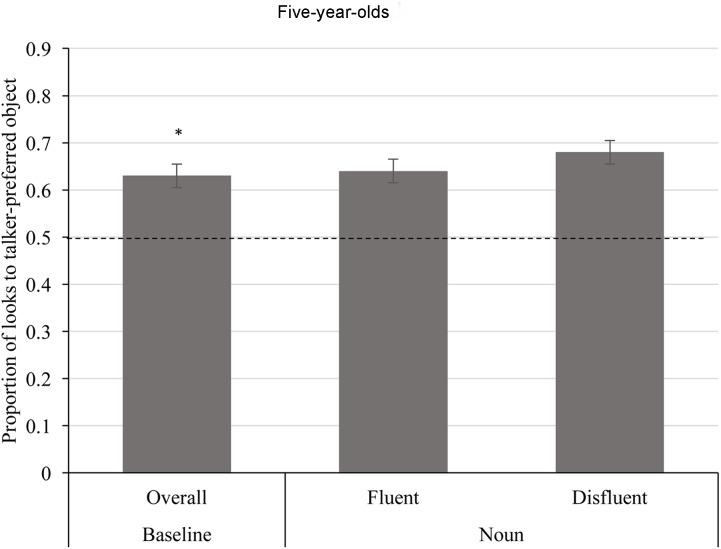
Average proportion of time looking to talker-preferred objects during the baseline interval and noun interval for 5-year-old age group. The average proportion of time looking to the talker-preferred object was significantly greater than chance (0.50) during the baseline interval (*p* < 0.001). There was no effect of fluency during the noun interval (*p* = 0.180). Error bars depict standard errors. A single asterisk represents a significant difference from chance (*p* < 0.05).

Again, we first compared the proportion of time looking to the talker-preferred objects to chance performance during the baseline interval, which allowed us to evaluate whether children held an expectation that talkers will refer to an object bearing their previously expressed preferred color. **Figure 4** (leftmost column) shows the average proportion of time looking to talker-preferred objects during the baseline interval. A one-sample *t*-test indicated that the proportion of time looking to the talker-preferred object (*M* = 0.63, *SD* = 0.09) during the baseline interval was significantly above chance levels (0.50), *t*(26) = 7.612, *p* < 0.001. This finding is consistent with the pattern found for adults, where looking patterns reflected an initial expectation for talkers to refer to talker-preferred objects as a sentence began to unfold.

#### Noun Interval

Next, we analyzed fixation patterns following the fluent/disfluent determiner to measure the impact on referential expectations. These data are shown in **Figure 4** (center and right columns) across the two fluency conditions. A repeated measures ANOVA revealed no significant effect of fluency (*p* = 0.180). Thus, children were not more likely to reduce consideration of the talker-preferred object during disfluent trials. In fact, children’s expectation that talkers would refer to their preferred object persisted throughout the noun interval for both fluent and disfluent trials. This is supported by the finding that the preference to fixate the talker-preferred object over the dispreferred object was significantly greater than chance (0.50) for both fluent utterances (*M* = 0.64, *SD* = 0.23), *t*(26) = 3.108, *p* = 0.005, and disfluent utterances (*M* = 0.68, *SD* = 0.22), *t*(26) = 4.214, *p* < 0.001.

The results of Experiment 2 indicate that 5-year-old children showed an increased proportion of looks to talker-preferred objects that began during the baseline interval and that remained unmoderated by the presence of a disfluency. Thus, children relied exclusively on the identity and associated gender-stereotyped color preference of the talker to form expectations about a speakers’ referential intent toward novel objects. To directly compare the results from the 5-year-olds to that of the adults, we conducted a two (age group: adults vs. 5-year-olds) × two (fluency: fluent vs. disfluent) mixed-model ANOVA on the data from the noun region. This analysis indicated a significant age by fluency interaction, *F*(1,48) = 7.021, $\eta_{p}^{2}$ = 0.128, *p* = 0.011, supporting the conclusion that adults and children differed in their use of the disfluency cue.

## General Discussion

The goal of the present study was to examine the effect that preference information and disfluency cues have on listeners’ expectations about talkers’ referential intent toward novel objects. In two experiments, we examined whether adults and children would use cues based on knowledge of talkers’ gender-stereotyped color preferences and talkers’ voice characteristics in conjunction with cues based on filled pause disfluencies to overcome referential uncertainty in the context of novel words in real time. The results revealed that while both adults and children initially relied on preference information when forming referential predictions, only adults can use disfluency cues to modify their earlier predictions.

The results of the present study broaden our understanding of preschool children’s skillful use of talkers’ preferences (reinforced by gender stereotypes) to interpret referential intentions. Instead of relying on desire statements (e.g., “*I want*…”), which explicitly signal that a talker’s preferences are relevant to the communicative context, we used utterances that were completely ostensive in nature in that they simply directed children to “look at” one of two novel objects in a display. The results indicated that talkers’ voices alone were sufficient to evoke children’s knowledge of talker-associated preference information, which in turn facilitated corresponding expectation of the potential referent of a novel word. Moreover, not only were children anticipating reference to talker-preferred objects, but they were doing so from the earliest moments of processing and well in advance of any label information. Taken together, it is clear that talker identity linked to preference information can serve as a highly effective cue to determine referential intent toward a novel object for both young children as well as mature language users.

Adults, on the other hand, were more judicious in the way in which they combined the cues involving talkers’ preferences with speech fluency cues in the course of forming referential expectations for novel words. Although adults also used their knowledge of talkers’ distinct color preferences, the hesitation disfluency in the unfolding utterance had the effect of dynamically updating adults’ expectations of the likely referent for the noun: When the noun was preceded by a filled pause disfluency, eye gaze data indicated that adults reduced their expectation that a talker was referring to a preferred novel object. This result in turn extends our understanding of the processing consequences associated with disfluent descriptions. As mentioned earlier, previous findings have shown that adults can use the disfluency–novelty link to infer referential intent, such that they will fixate a novel referent over a familiar referent upon hearing a filled pause (e.g., Owens et al., 2017). Yet both objects in the present study were equally unfamiliar to adult (and child) listeners. One possibility is that talker-specific preference information was used as a proxy for the talker’s level of familiarity with the objects. That is, adult listeners may have assumed that the talkers were more familiar with the novel objects bearing their gender-stereotyped preferred color, compared to those bearing their non-preferred color. Then, when a filled pause was encountered, this may have activated adults’ previously acquired disfluency-novelty link, which then led them to reduce their consideration of the preferred-colored object that would be more familiar to the talker. This explanation is in line with previous research demonstrating that filled pauses lead adult listeners to infer plausible reasons for the delay by taking the talker’s perspective (e.g., Barr and Seyfeddinipur, 2010), and do not simply rely on well-worn associations about the distribution of disfluencies in speech. In addition, this finding contributes to a broadened understanding of how exactly filled pauses achieve their effects. Specifically, not only can filled pauses direct attention toward novel referents (e.g., Barr, 2001; Arnold et al., 2007), they can also serve as a cue to shift away from predictions that are otherwise promoted by the available cues (e.g., Corley et al., 2007; MacGregor et al., 2010).

In contrast, children did not use speech fluency cues to revise their hypotheses when forming referential predictions. This finding was somewhat surprising, given that recent research has demonstrated that 5-year-old children can indeed engage these same two cues during language processing in other contexts (Thacker et al., 2018). However, an important difference between these two paradigms is that in Thacker et al. (2018) the referents were familiar, whereas the present study used novel objects and labels. If disfluencies in the present study signaled that the talker was likely to refer to the unfamiliar-to-them object (i.e., the talker-non-preferred object), then it is understandable that children did not use the disfluency cue, as this result then echoes previous findings suggesting that children of this age do not associate disfluency with unfamiliarity alone (Owens et al., 2017). Relatedly, disfluencies in the present study may have implicitly conveyed to children that the talker was unsure whether she was using the correct object label. Since previous research has shown that children will avoid learning words from uncertain talkers (Sabbagh and Baldwin, 2001), it follows that children in the present study would avoid mapping the novel word onto the (potentially) erroneous non-preferred object. Regardless of the precise interpretation, our results suggest that 5-year-olds weighted talkers’ preferences as more relevant for identifying the referent of a novel noun phrase, compared to the fluency of their speech.

In sum, our results contribute to a growing body of research investigating how children’s appreciation of distinct mental states contributes to the process of determining referential intent. Our findings demonstrate that in some cases, young word learners will rely on one talker-producued cue, in this case preference information, when forming referential expectations for novel word. Adults, on the other hand, demonstrated a sophisticated ability to rapidly amend initial referential predictions in response to newly encountered information in the context of novel words.

## Ethics Statement

This study was carried out in accordance with the recommendations of University of Calgary Conjoint Faculties Research Ethics Board (CFREB) with written informed consent from all participants. The protocol was approved by the University of Calgary Conjoint Faculties Research Ethics Board (CFREB).

## Author Contributions

JT conducted this research in partial fulfillment on the requirements for the M.Sc. degree, under the supervision of SG. All authors contributed to the conceptualization, design, and analysis of the data and to the preparation of the manuscript.

## Conflict of Interest Statement

The authors declare that the research was conducted in the absence of any commercial or financial relationships that could be construed as a potential conflict of interest.
